# A Rare Case of Esophageal Leukoplakia: A Potential Precursor to Esophageal Malignancy

**DOI:** 10.7759/cureus.17205

**Published:** 2021-08-15

**Authors:** Muhammad Farhan Ashraf, Seth Richter, Soe H Arker, Nour Parsa

**Affiliations:** 1 Internal Medicine, Albany Medical Center, Albany, USA; 2 Gastroenterology and Hepatology, Albany Medical Center, Albany, USA; 3 Pathology, Albany Medical Center, Albany, USA

**Keywords:** endoscopy, carcinogenesis, esophageal leukoplakia, esophageal epidermoid metaplasia, endoscopic mucosal ablation

## Abstract

Esophageal leukoplakia or epidermoid metaplasia is a rare lesion resembling the commonly found oral leukoplakia. When found, it is typically seen incidentally on endoscopy as a white plaque but rarely it may present as a globus sensation. Histologically, it is seen as epidermal metaplasia with orthokeratosis, closely resembling the skin. Although rare, esophageal leukoplakia is precancerous and may pose a serious threat.

We present a unique case of a 61-year-old male with a history of COPD, tobacco, and alcohol dependence presenting with a six-month history of nausea and emesis resulting in poor oral intake despite having an appetite. The patient also reported weight loss. Considering his risk factors for esophageal carcinoma and alarm symptoms, an upper endoscopy was performed that revealed localized white, plaque-like mucosal changes characterized by altered texture in the lower third of the esophagus at 40cm. Biopsy results showed squamous epithelium with orthokeratosis and a prominent granular cell layer. These findings were consistent with esophageal epidermoid metaplasia. The lesion was ablated using argon plasma coagulation and radiofrequency ablation on subsequent endoscopy. The patient reported continued resolution of symptoms with each treatment session.

Esophageal leukoplakia may increase the risk for squamous cell carcinoma of the esophagus and should be followed closely. Guidelines on surveillance are yet to be established given the rarity of the disease.

## Introduction

Leukoplakia is a term describing a white patch or plaque on mucous membranes that cannot be rubbed off easily and cannot be clinically characterized as any other disease [1]. Esophageal epidermoid metaplasia, also known as esophageal leukoplakia, is an extremely rare precancerous lesion that histologically resembles the epidermis of the skin. It is related to oral leukoplakia and has the potential to transform into squamous cell carcinoma [2]. One study found a prevalence of 0.19% in a group of 1048 patients who underwent esophageal biopsies (for a variety of indications) [3]. It can rarely present with symptoms of dysphagia or globus sensation and is typically an incidental finding [4]. On endoscopy, esophageal epidermoid leukoplakia presents as a scaly white plaque with clear borders. Histologically, it is identified by the presence of epidermal metaplasia, a prominent granular layer, orthokeratosis, and an abrupt transition from surrounding squamous epithelium [5]. We present the case of a 61-year-old male with esophageal leukoplakia who presented with nausea, vomiting, and solid food dysphagia.

## Case presentation

A 61-year-old male with a past medical history of COPD, tobacco, and alcohol use presented with a six-month history of nausea, emesis, and frequent episodes of solid food dysphagia. The patient also endorsed five pounds of unintentional weight loss during this period that he attributed to his apprehension to eat despite reporting a good appetite. His social history was notable for drinking a six-pack of beer daily for the last 40 years in addition to a 40 pack-year smoking history. The patient denied any abdominal pain, fevers, chills, or diarrhea. The patient denied any personal or family history of diabetes, malignancy, or motility disorders. He had no known history of being in an immune-compromised state. His vitals were stable and his physical examination was normal.

**Figure 1:**
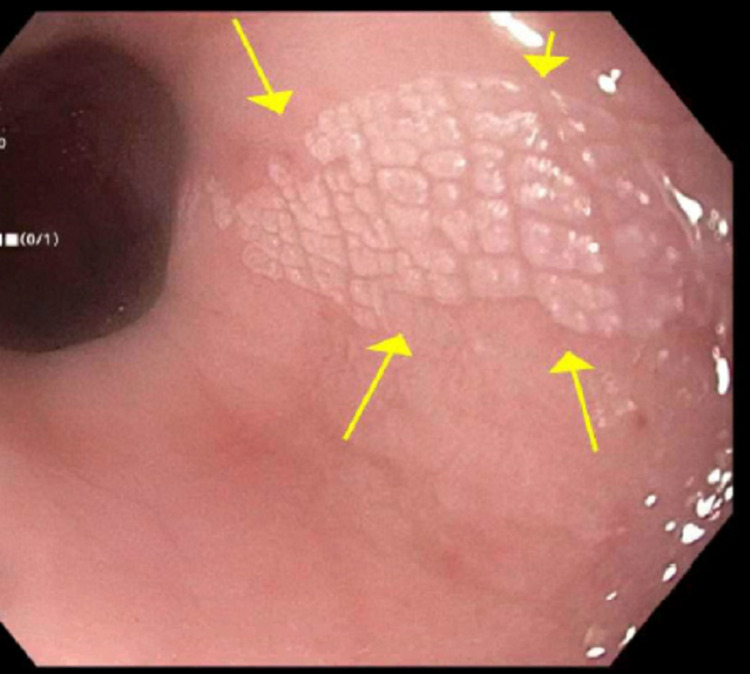
Endoscopic view of the esophageal plaque

On laboratory examination, his complete blood count (CBC) revealed a white blood cell count of 10200/uL, hemoglobin of 15.3 g/dL, hematocrit of 44 %, and platelet count of 125000 /uL. Erythrocyte sedimentation rate (ESR) and C-reactive protein (CRP) were elevated at 38 mm/hr and 100.4 mg/L respectively. Computed tomography (CT) of the chest showed secretions and diffuse bronchial wall thickening consistent with aspiration-related bronchiolitis. It also showed circumferential distal esophageal thickening. An EGD (Esophagogastroduodenoscopy) was performed that showed white plaque-like mucosal changes characterized by altered texture found in the lower third of the esophagus at 40cm (figure 1).

**Figure 2:**
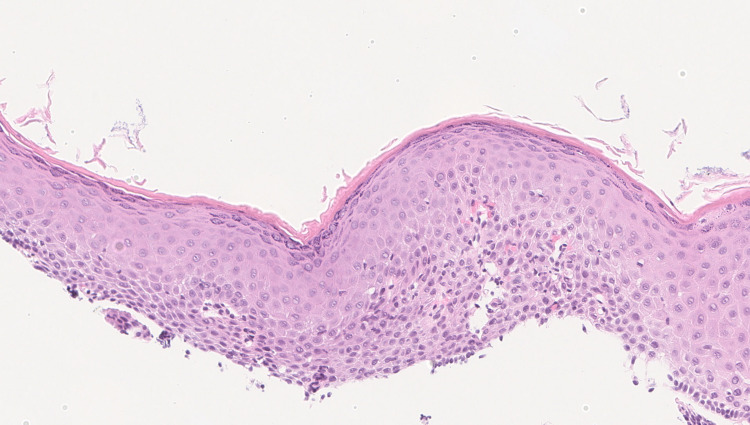
Slide showing epidermoid metaplasia (Squamous epithelium with orthokeratosis and a prominent granular cell layer)

Biopsies were taken with cold forceps for histology and revealed squamous epithelium with orthokeratosis and a prominent granular cell layer. These findings were consistent with esophageal epidermoid metaplasia, a rare lesion analogous to oral leukoplakia (figure 2).

Differential diagnosis before endoscopy included malignancy versus a benign stricture, esophageal web, or a motility disorder. The patient had more dysphagia with solids than liquids. Also, his presentation was subacute. These points went against a motility disorder. No stricture was noticed on the endoscopy. Biopsy of the plaque-like lesion found on EGD confirmed the final diagnosis of esophageal leukoplakia.

**Figure 3:**
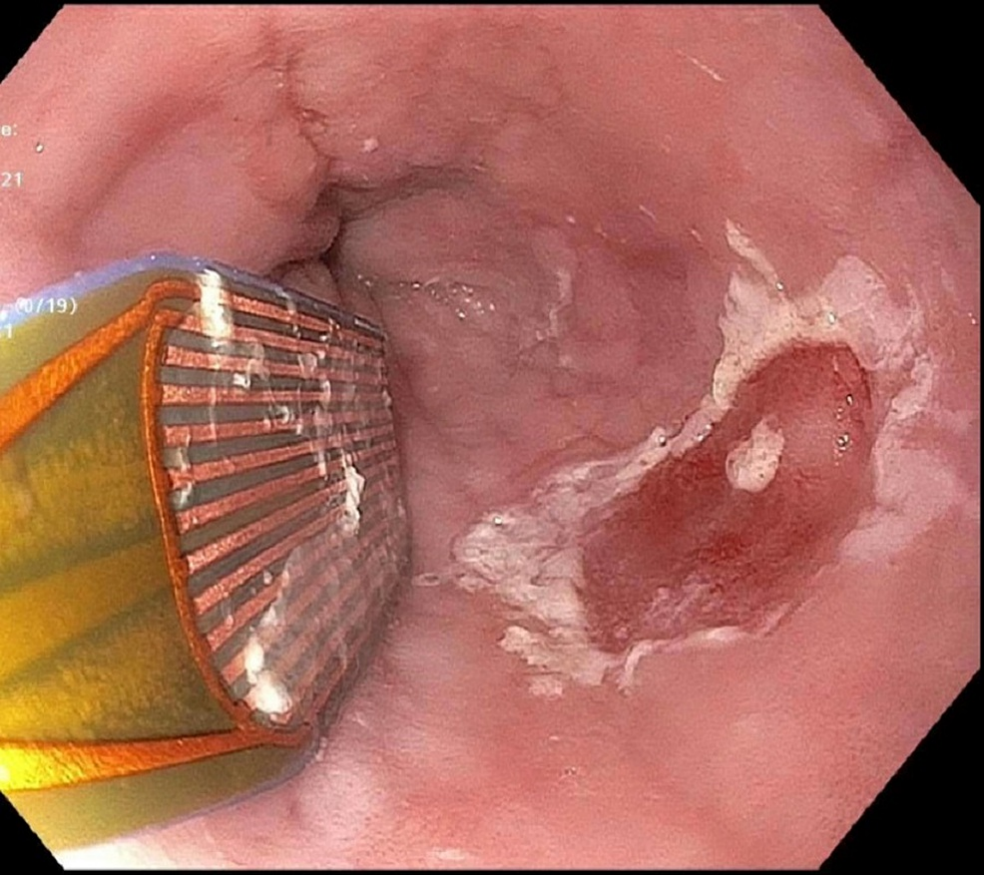
Radiofrequency ablation of the residual lesion

The patient underwent a repeat endoscopy, and the lesion was ablated using argon plasma coagulation (APC). The patient was advised to abstain from alcohol and tobacco consumption. He was also started on omeprazole. He reported progressive improvement in symptoms. Three months later, a repeat endoscopy was performed to confirm the resolution of esophageal leukoplakia. This endoscopy showed a 2mm residual lesion which was completely ablated using radiofrequency ablation (figure 3).

On follow-up, he reported only 1-2 episodes of dysphagia over a period of two months (compared to previous symptoms of multiple episodes weekly). He is scheduled for a repeat endoscopy in six months for surveillance and is working under the direction of his primary care physician to taper his tobacco and alcohol use.

## Discussion

Oral leukoplakia is a relatively common and painless lesion of the oral mucosa. It has a strong male predominance and is associated with tobacco and alcohol use. In contrast, esophageal leukoplakia or esophageal epidermoid metaplasia is an extremely rare condition and is more common in females. One series showed only six cases in 1000 autopsy specimens and another study showed only two cases in 1048 consecutive biopsies [3, 6]. Taggart et al. proposed that the risk factors for oral and esophageal leukoplakia were similar. Tobacco and alcohol are the most common risk factors. Although HPV (Human Papilloma Virus) is a risk factor for oral leukoplakia, no such association has been established with esophageal leukoplakia.

The etiology of esophageal leukoplakia is currently unknown. Chronic irritation from acid reflux is proposed to be the likely cause although Barrett’s esophagus and remains the most common metaplasia related to acid reflux [7]. Although most of the esophageal epidermoid metaplasia cases in the literature were reported incidentally in asymptomatic patients, some people did present with dysphagia as the predominant symptom [8].

On endoscopy, esophageal leukoplakia is usually seen as a superficially raised white or scaly plaque above the squamocolumnar junction as demonstrated in our case [9]. Mucosal plaques are common in the esophagus and are most commonly secondary to glycogenic acanthosis. These plaques are composed of hyperplastic squamous epithelium with intracellular glycogen deposits [10]. Other plaque-forming lesions include esophageal infections, squamous cell papillomas, hyperkeratosis, and plaques from reflux esophagitis. In contrast to the above-mentioned lesions, esophageal leukoplakia has a distinct squamous epithelium with a prominent granular cell layer and a layer of hyperorthokeratosis resembling the epidermis of the skin.

Interestingly, like squamous cell carcinoma, esophageal epidermoid metaplasia is not stained by Lugol's iodine making it distinct from the aforementioned lesions. It is a precursor to squamous cell carcinoma and can sometimes be seen adjacent to a high-grade squamous cell carcinoma [8]. Targeted next-generation sequencing supports epidermoid metaplasia of the esophagus as a precursor to esophageal squamous neoplasia. Detection of multiple mutations including TP53, which are associated with esophageal squamous cell carcinoma, in esophageal epidermoid metaplasia, corroborates the clonal relationship between these entities [2,11].

Treatment is mainly the destruction of lesions with either radiofrequency ablation or argon plasma coagulation. In contrast to other more common precursor metaplastic lesions such as Barrett’s esophagus, there are no current guidelines available for post-ablation surveillance of esophageal leukoplakia. We performed a surveillance endoscopy three months after the first ablation. In our case multiple ablations were required due to residual lesion found on repeat endoscopy. Some clinicians propose annual endoscopic evaluation with four-quadrant biopsies at every 1cm of the affected area [12]. More studies are needed to establish universal surveillance guidelines.

## Conclusions

Esophageal leukoplakia is a rare and under-reported lesion that may progress to esophageal squamous cell carcinoma. It may infrequently present as dysphagia and a globus sensation. Esophageal leukoplakia should be considered in the differential diagnosis of esophageal plaques found on EGD. Endoscopic ablation is the preferred treatment and multiple endoscopies may be required to completely eradicate this lesion as in our case. Guidelines should be developed for post-ablation surveillance as no such guidelines exist.
